# Epigenetic modification of miR-141 regulates SKA2 by an endogenous ‘sponge’ HOTAIR in glioma

**DOI:** 10.18632/oncotarget.8895

**Published:** 2016-04-21

**Authors:** Er-Bao Bian, Chun-Chun Ma, Xiao-Jun He, Chao Wang, Gang Zong, Hong-Liang Wang, Bing Zhao

**Affiliations:** ^1^ Department of Neurosurgery, The Second Affiliated Hospital of Anhui Medical University, 230601, Hefei, China; ^2^ Cerebral Vascular Disease Research Center, Anhui Medical University, 230601, Hefei, China

**Keywords:** gliomas, miR-141, HOTAIR, SKA2, DNMT1

## Abstract

Aberrant expression of miR-141 has recently implicated in the occurrence and development of various types of malignant tumors. However whether the involvement of miR-141 in the pathogenesis of glioma remains unknown. Here, we showed that miR-141 was markedly downregulated in glioma tissues and cell lines compared with normal brain tissues, and its expression correlated with the pathological grading. Enforced expression of miR-141 in glioma cells significantly inhibited cell proliferation, migration and invasion, whereas knockdown of miR-141 exerted opposite effect. Mechanistic investigations revealed that HOTAIR might act as an endogenous ‘sponge’ of miR-141, thereby regulating the derepression of SKA2. Further, we explored the molecular mechanism by which miR-141 expression was regulated, and found that the miR-141 promoter was hypermethylated and that promoter methylation of miR-141 was mediated by DNMT1 in glioma cells. Finally, both overexpression of miR-141 and knockdown of HOTAIR in a mouse model of human glioma resulted in significant reduction of tumor growth *in vivo*. Collectively, these results suggest that epigenetic modification of miR-141 and the interaction of ceRNA regulatory network will provide a new approach for therapeutics against glioma.

## INTRODUCTION

Malignant gliomas are the most common primary tumors of the brain and are divided into four histopathologic grades based on the degree of malignancy [1]. Glioblastoma multiforme (GBM), known as grade IV glioma, is characterized by highly invasive growth pattern, and is often related to high incidences of postresection recurrence and poor prognosis [2]. GBM invasion comprises a series of discrete biological processes, including detachment of tumor cells from the original site, and a multistep cascade of coordinated cell adhesion and contractility as well as a proteolytic remodeling of the extracellular matrix [3]. Although various cells and molecules are available on malignant process, a critical need still exists to identify novel targets that can be used to curb the progression of glioma.

MicroRNA (miRNA) is emerging as a new paradigm in the field of glioma. MiRNAs are highly conserved, single stranded, non-coding RNAs of approximately 22 nucleotides that can downregulate various gene products [4].

MiRNA is preferentially incorporated into an enzyme complex named RNA-induced silencing complex (RISC), which composed of Dicer, TRBP, and a protein of the Argonaute (Ago) superfamily. Within the help of this complex, miRNAs cause target cleavage or translation repression after binding to targets by complementary base pairing within the 3-untranslated region [4, 5].

Therefore, miRNAs regulate diverse cellular processes, including cell-cycle progression, proliferation, invasion and development, and function as oncogenes or tumor suppressor miRNAs [6, 7]. Dysregulated expression of miRNAs has been linked to various kinds of cancer, such as gliomas [8, 9]. MiRNA dysregulation can be regulated by many factors, including epigenetic alterations such as DNA methylation, and histone modification [10, 11]. MiR-141 is a member of the miR-200 family that can affect malignant phenotype of tumor cells [12]. Recently, epigenetic modification of miR-141 has been detected in breast cancer cell, correlating with their downregulation [13]. However, the exact role of miR-141 in gliomas has not yet been elucidated.

Long non-coding RNAs (LncRNAs) are transcribed RNA molecules more than 200 nucleotides in length, but they have no significant protein-coding potential [14]. LncRNAs regulate the expression of genes at the epigenetic, transcriptional and post-transcriptional levels, and play an important role in physiological processes [15]. LncRNAs are involved in a variety of biological processes, including proliferation, invasion, differentiation, and chromatin modification through multiple regulation of mechanisms [16]. Recently, a novel regulatory mechanism has been found in which the interaction between lncRNAs and mRNAs occurs through competing for shared miRNAs response elements [17]. Hox transcript antisense intergenic RNA (HOTAIR), well known as a lncRNA, is located on chromosome 12q13.13, which has a functional role in trans-silencing [18]. HOTAIR expression levels are highly in diverse types of cancers, including breast, colon and liver cancer [19–21]. In addition, HOTAIR is closely associated with glioma grade, as well as a critical regulator of cell cycle progression [22]. To date, emerging evidence has strongly suggested that aberrant lncRNA expression is a feature of human glioma. However, miRNA-lncRNA network in glioma remains unknown and needs further investigation.

In this study, we provide evidence for the role of miR-141 as one of the tumor-suppressive miRNAs that is frequently downregulated in glioma samples and cell lines, through methylation of its promoter. Interestingly, HOTAIR may act as a ‘sponge’ of miR-141, thereby modulating expression of SKA2 in glioma. The present work provides specific epigenetic regulatory network between methylation, miRNA, and lncRNA, suggesting a novel strategy for the prevention and treatment of gliomas based on targeting miR-141 and its downstream HOTAIR.

## RESULTS

### The loss of miR-141 expression in gliomas

**Figure 1:**
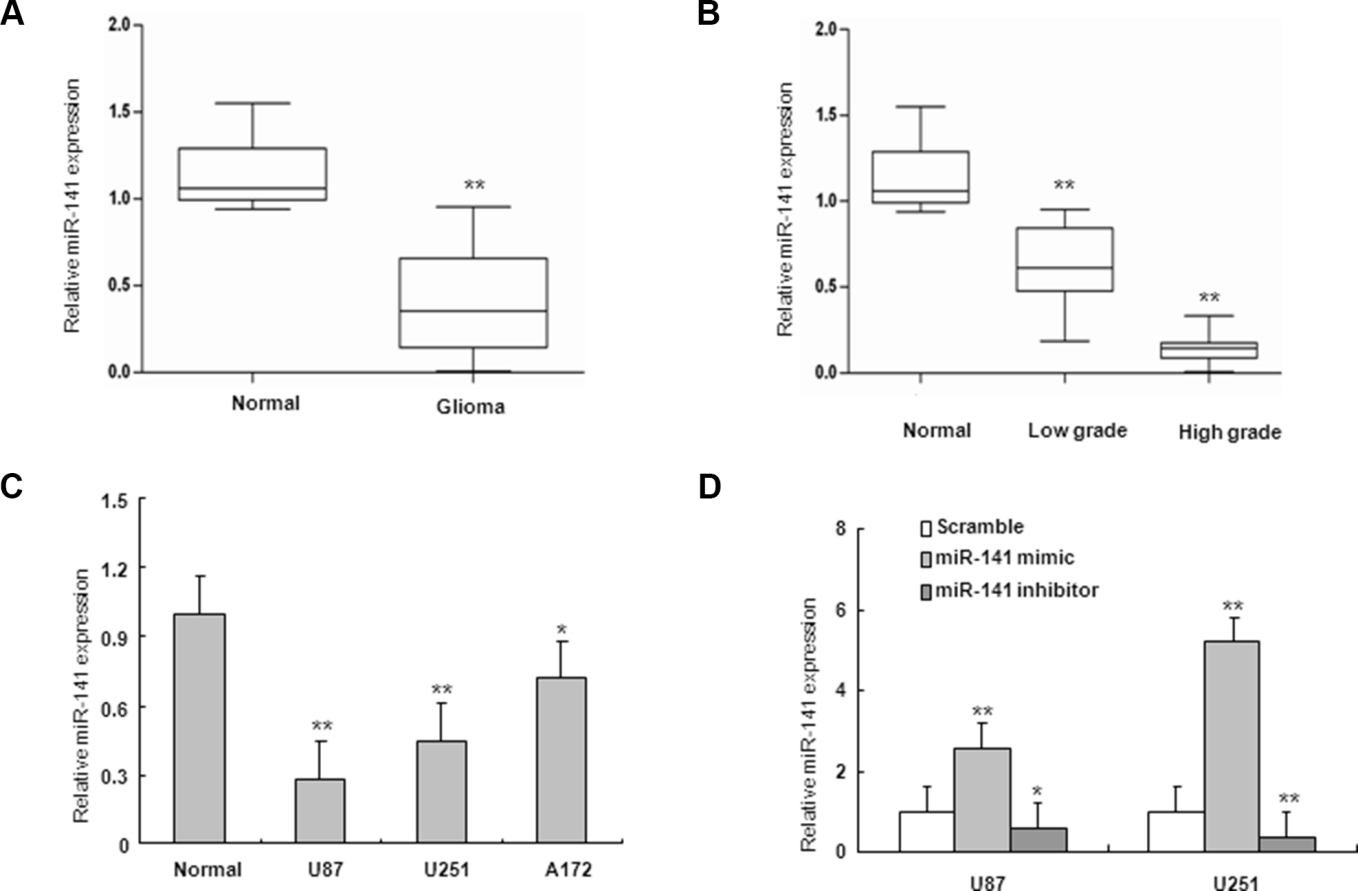
miR-141 is downregulated in glioma samples and glioma cells (**A**) miR-141 expression in glioma and normal brain tissues by qRT-PCR. (**B**) qRT-PCR analysis of miR-141 in different grade glioma and normal brain tissues.(**C**) qRT-PCR analysis showed that U87, U251, and A172 glioma cells express low levels of miR-141, compared with normal brain tissues. (**D**) qRT-PCR analyses of the inhibition or overexpression efficiencies of miR-141 in U87 and U251 glioma cells. The data represent the mean ± SD of three different experiments.*P < 0.05, **P < 0.01 versus Normal or scramble control.

To determine the levels of miR-141 in glioma samples and cell lines, total RNAs were extracted from glioma tissues at grades I, II, III, and IV, normal brain tissue samples and glioma cell lines, and the expression levels of miR-141 were analyzed using qRT-PCR. As shown in Figure 1A, the levels of miR-141 expression in glioma tissues (*n* = 56) were significantly down-regulated compared with normal brain tissues (*n* = 11). To explore whether there is any association between the loss of miR-141 and the pathogenesis of glioma, we detected the expression of miR-141 in glioma tumor samples with different histopathologic grades and found a significant decrease in low grade glioma samples, whereas a much stronger decrease was observed in high grade glioma samples, suggesting that miR-141 may be involved in pathogenesis of glioma (Figure 1B). It was also shown that miR-141 was down-regulated in 3 glioma cell lines, compared with 6 normal brain tissues (Figure 1C). Taken together, our results revealed that miR-141 was abnormally down-regulated in glioma and its expression level negatively correlated with disease severity.

### MiR-141 inhibits glioma cells proliferation, migration and invasion

**Figure 2:**
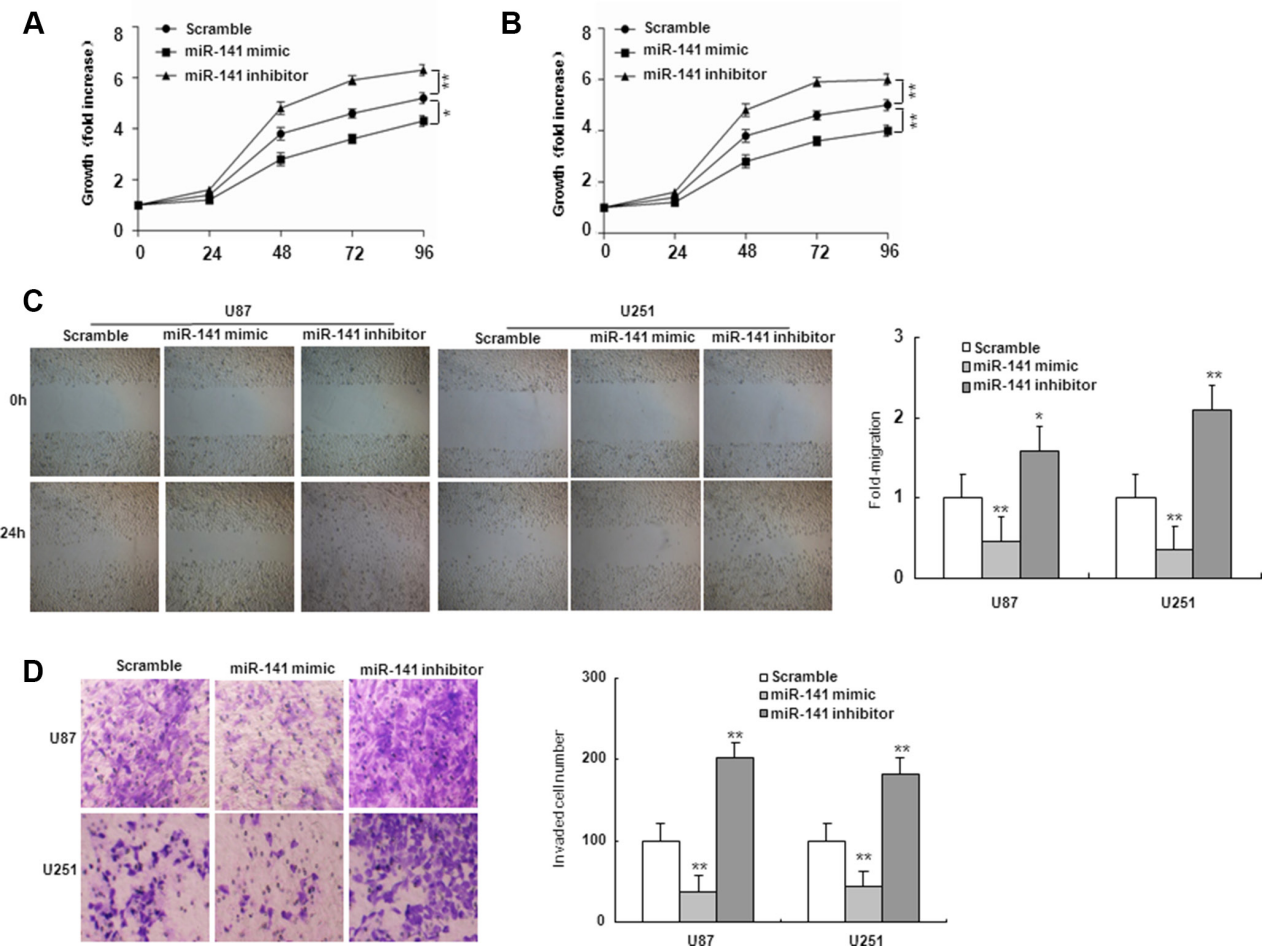
miR-141 suppresses cell proliferation, migration, and invasion in U87 and U251 glioma cells (**A**, **B**) Effects of miR-141 on cell proliferation in U87 and U251 glioma cells analyzed using the MTT assay.(**C**, **D**) Effects of miR-141 on cell migration, and invasion in U87 and U251 glioma cells analyzed using Wound-healing assay and Transwell invasion assay, respectively. The data represent the mean ± SD of three different experiments. *P < 0.05, **P < 0.01 versus scramble control.

To investigate the role of miR-141 in glioma, U251 and U87 glioma cells were transiently transfected with miR-141 mimic, miR-141 inhibitor or scramble. QRT-PCR analyses showed that miR-141 was significantly increased in cells transfected with miR-141 mimic and decreased in the miR-141 inhibitor-transfected group compared with scramble (Figure 1D). MTT assay showed that miR-141 mimic significantly inhibited cells proliferation both in U251 and U87 cells, whereas miR-141 inhibitor promotes cell growth in both cell lines (Figure 2A–2B). The wound healing assay showed that miR-141 overexpression exhibited considerably slower migration and reduced cell spreading of both U87 and U251 cells, whereas miR-141 knockdown boosted cell migration in both cell lines (Figure 2C). To test whether miR-141 expression influences the invasive behavior of U87 and U251 glioma cells, we performed transwell assays. Overexpression of miR-141 significantly inhibited the invasion of cells, whereas the knockdown of miR-141 enhanced cells invasion (Figure 2D). Collectively, these results indicated that miR-141 strongly inhibited the proliferation and invasion of glioma cells.

### MiR-141 is able to directly repress and bind to HOTAIR

**Figure 3:**
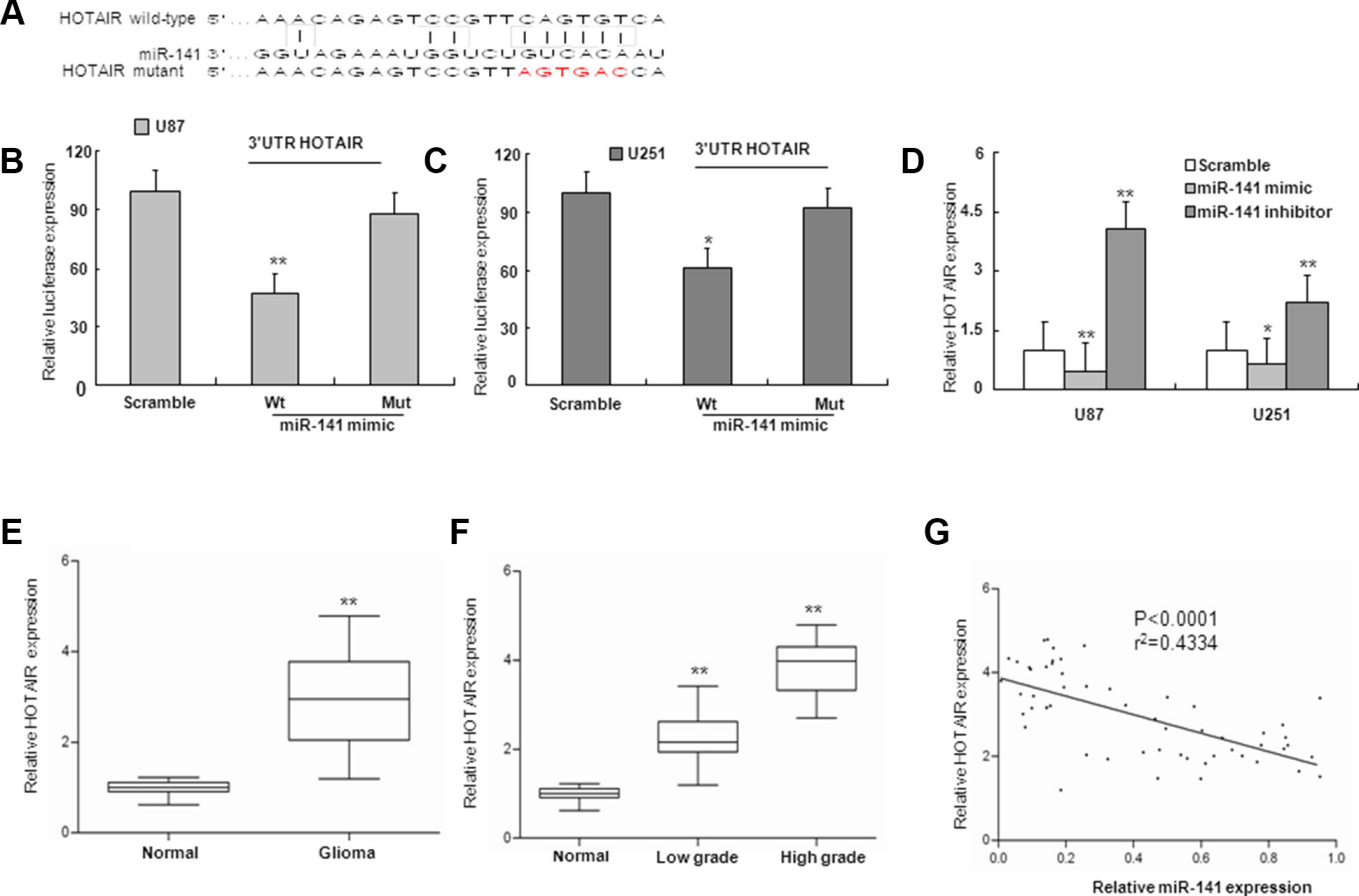
miR-141 targets and negatively is correlated with HOTAIR (**A**) miR-141 binding sequence in HOTAIR. The red letters in HOTAIR sequence are mutated. (**B**, **C**) miR-141 mimic repressed Luciferase activity of the wild but not the mutant 3′UTR of HOTAIR reporter in U87 and U251 glioma cells (**D**). The expression of HOTAIR mRNA was observed after transfection with miR-141 mimic or miR-141 inhibitor. (**E**) The expression of HOTAIR in glioma samples and normal brain tissues by qRT-PCR. (**F**) qRT-PCR analysis of HOTAIR in different grade glioma and normal brain tissues (**G**). The correlation between miR-141 and HOTAIR mRNA level was measured in 56 glioma tissues. The ΔCt values were subjected to Pearson correlation analysis. The data represent the mean ± SD of three different experiments.*P < 0.05, **P < 0.01 versus Normal or scramble control.

MiRNAs exert their functions by targeting multiple transcripts that are coordinately orchestrated in a biological process. By using similar strategies as described in previous study [23], HOTAIR was predicted as one of the targets of miR-141 (Figure 3A). To validate whether HOTAIR is target of miR-141 in U87 and U251 glioma cells, we constructed luciferase reporter plasmid containing 3′UTR for HOTAIR. As shown in Figure 3B–3C, ectopic expression of miR-141 markedly decreased the reporter luciferase activities. Furthermore, mutagenesis in miR-141 target sites of the HOTAIR 3′UTR linked to the luciferase reporter confirmed the site-specific effect of miR-141 (Figure 3B–3C). MiR-141–mediated regulation of HOTAIR expression in U87 and U251 glioma cells was further verified by HOTAIR mRNA expression analysis using qRT-PCR. HOTAIR mRNA expression was markedly decreased by transfection of miR-141 mimic, and was upregulated by transfecting miR-141 inhibitor in U87 and U251 glioma cells (Figure 3D). Hence, the downregulation of HOTAIR by miR-141 was directly dependent on the recognition site in the HOTAIR 3′UTR in glioma cells.

### HOTAIR expression is inversely correlated with miR-141 in human glioma tissues

Because miR-141 could repress the expression of HOTAIR, we investigated whether an inverse relationship existed between miR-141 expression and levels of HOTAIR. We examined expression of HOTAIR mRNA in human glioma samples. The HOTAIR mRNA levels were significantly up-regulated in glioma samples in comparison with normal brain tissues, and the upregulation of HOTAIR associated with glioma malignancy (Figure 3E–3F). Next, we investigated whether HOTAIR mRNA expression was inversely correlated with levels of miR-141 in glioma tissues. A total of 56 glioma tissues were analyzed for the expression levels of HOTAIR mRNAs and for miR-141 expression by qRT-PCR. The correlation of low miR-141 expression with high HOTAIR expression in human glioma patients is consistent with our finding that overexpression of miR-141 can downregulate HOTAIR in glioma cells (Figure 3G).

### MiR-141 regulates glioma cells proliferation and invasion in part by HOTAIR

**Figure 4:**
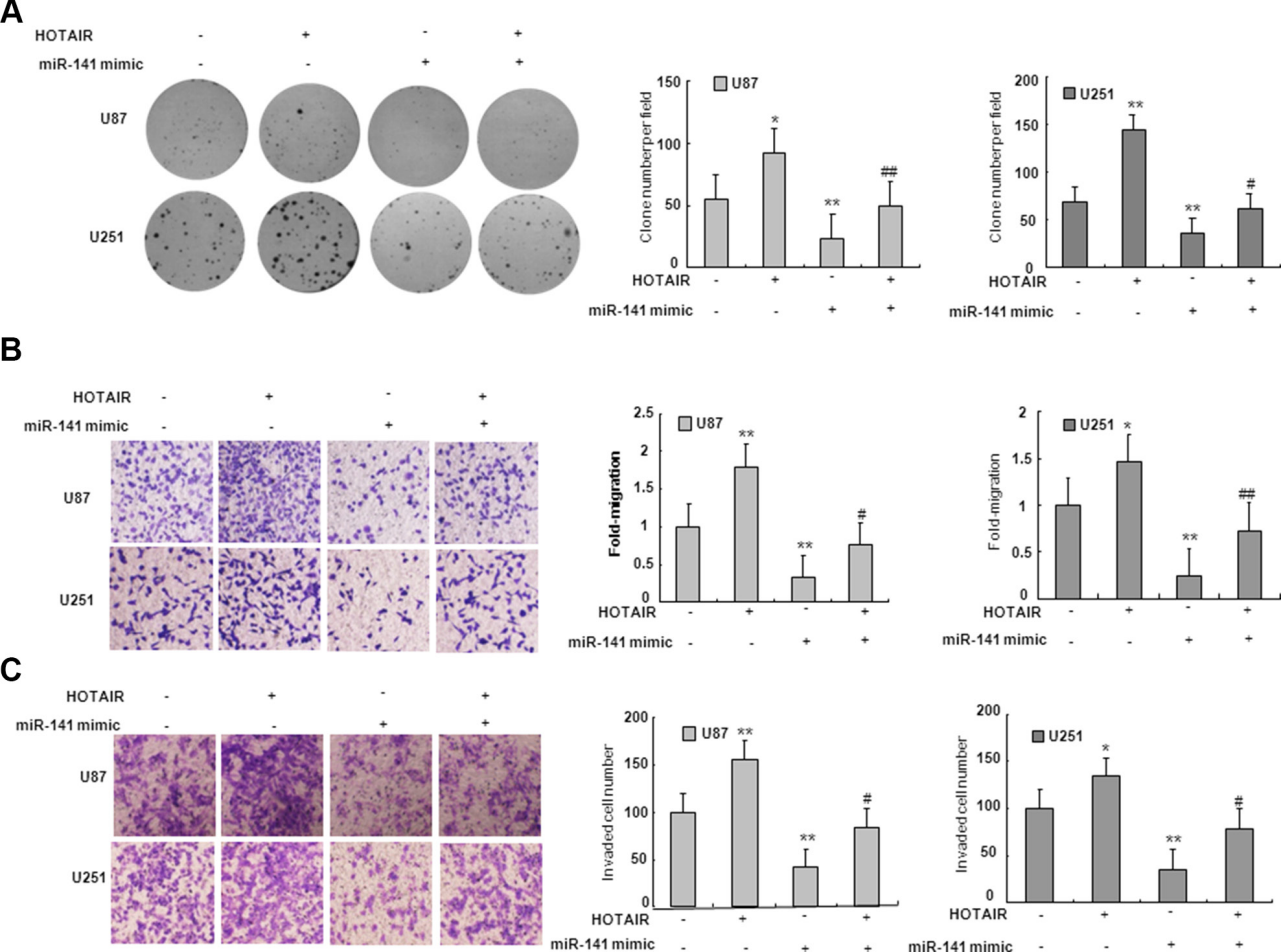
miR-141's antitumor activity is in part through negative regulation of HOTAIR (**A**) HOTAIR restores clone formation which is suppressed by miR-141 mimic in U87 and U251 glioma cells. (**B**) HOTAIR restores cell migration which is inhibited by miR-141 mimic in U87 and U251 glioma cells.(**C**) HOTAIR restores cell invasion which is inhibited by miR-141 mimic in U87 and U251 glioma cells. The data represent the mean ± SD of three different experiments. *P < 0.05, **P < 0.01 versus scramble control.

To examine if the effect of miR-141 on cell proliferation and invasion is mediated by HOTAIR, we co-transfected U87 and U251 cells with HOTAIR plasmid and miR-141 mimic. We demonstrated that miR-141 mimic suppressed the clone formation of glioma cells while HOTAIR reversed the decrease in clone formation (Figure 4A). As shown in Figure 4B, Transwell assays analysis indicated that miR-141 mimic inhibited the migration of glioma cells while HOTAIR rescued the migration. Similarly, the inhibition of glioma cells invasion mediated miR-141 mimic was in part rescued HOTAIR (Figure 4C). These results may imply that the tumor-suppressive function of miR-141 is partly through negative regulation of HOTAIR in glioma.

### SKA2 is a direct target miR-141

**Figure 5:**
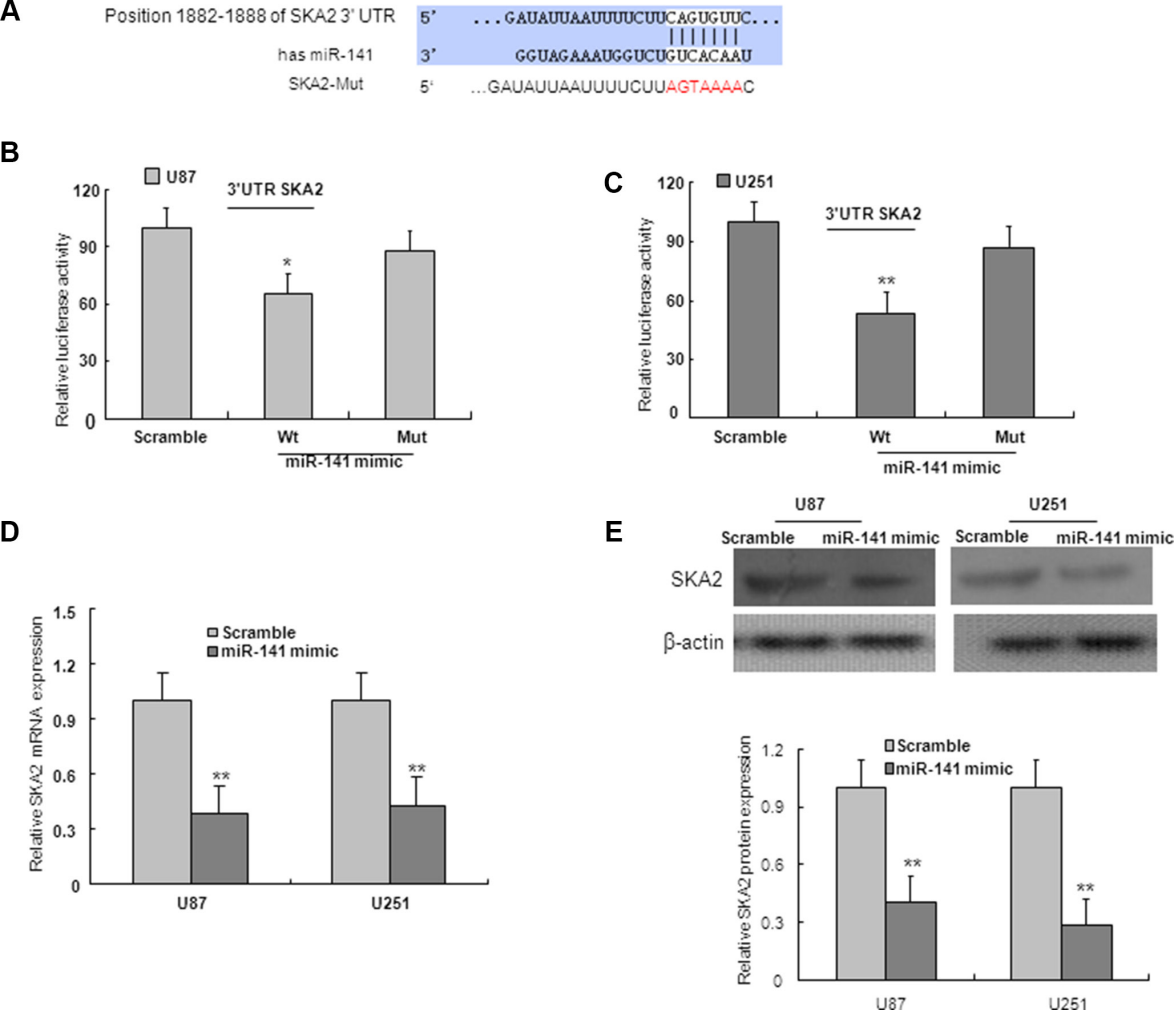
SKA2 is downstream target of miR-141 (**A**) Predicted binding of miR-141 to the 3′UTR of human SKA2 by TargetScan. (**B**, **C**) miR-141 mimic repressed Luciferase activity of the wild but not the mutant 3′UTR of SKA2 reporter in U87 and U251 glioma cells. (**D**) SKA2 mRNA expression was downregulated after transfection with miR-141 mimic in U87 and U251 glioma cells. (**E**) miR-141 mimic decreased the expression of SKA2 protein in U87 and U251 glioma cells. The data represent the mean ± SD of three different experiments.*P < 0.05, **P < 0.01 versus scramble control.

**Figure 6:**
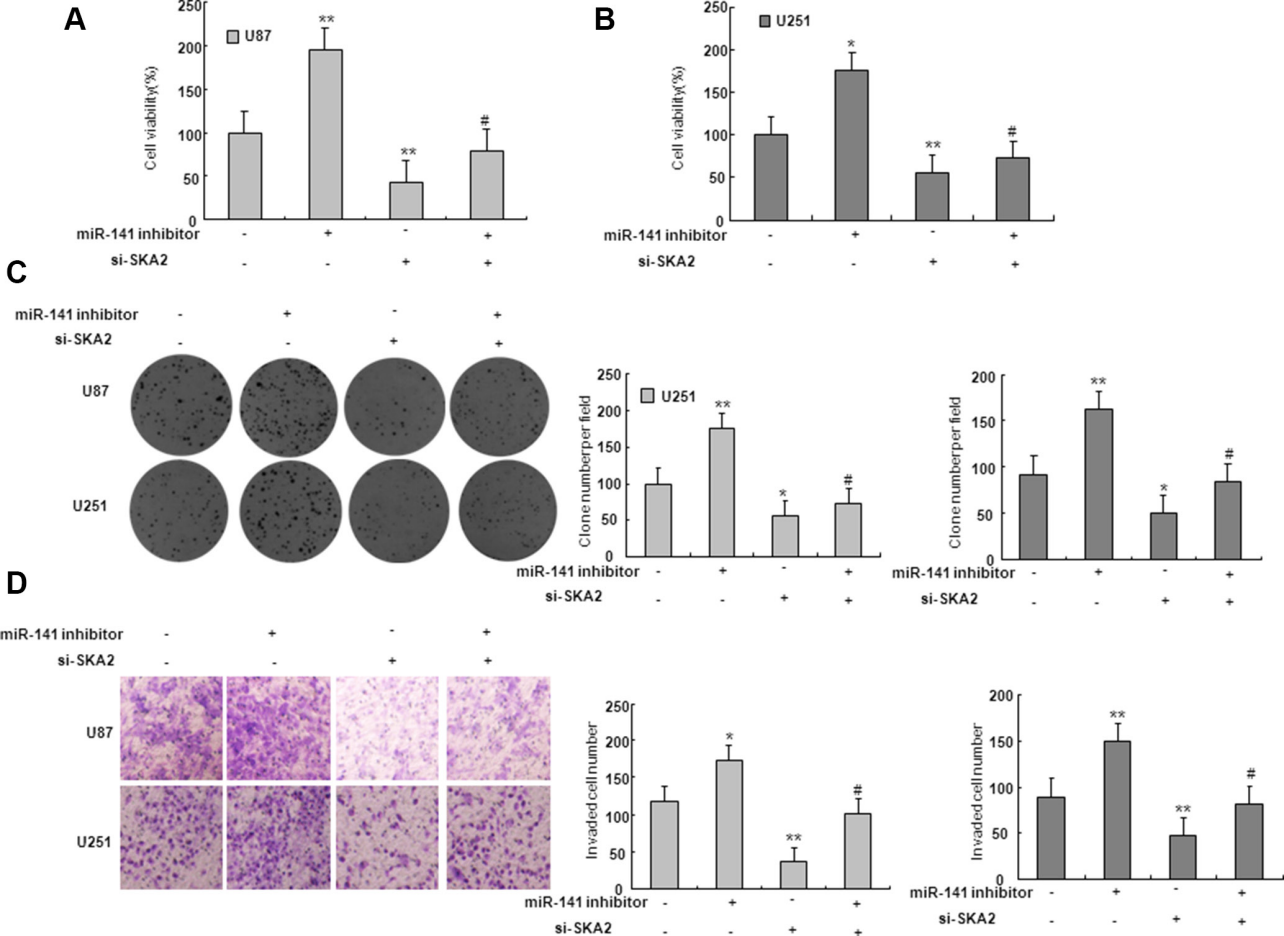
SKA2 mediated the effect of miR-141 on glioma cells (**A**–**B**) MTT assay was performed to detect the proliferation after co-transfected with miR-141 inhibitors and si- SKA2. *P < 0.05, **P < 0.01 vs. control group; ^#^*P* < 0.05 vs. miR-141 inhibitors group. (**C**) Colony formation assay was performed to determine the proliferation effect of glioma cells after co-transfected with miR-141 inhibitors and si- SKA2. *P < 0.05, **P < 0.01 vs. control group; ^#^*P* < 0.05 vs. miR-141 inhibitors group. (**D**) Invasion assay in glioma cells was performed to determined cell invasiveness after co-transfected with miR-141 inhibitors and si- SKA2. **P < 0.01 vs. control group; ^#^*P* < 0.05 vs. miR-141 inhibitors group. Data are presented as mean ± SD from three independent experiments.

On the basis of miRNA target prediction databases such as TargetScan, miR-141 has the predicted seed matches in the 3′UTR of SKA2 (Figure 5A). To verify that SKA2 is a direct target of miR-141 in U87 and U251 cells, the 3′UTR of SKA2 with wild type or mutant seed sequence recognizing sites was cloned to a dual-luciferase reporter. After co-transfection of miR-141 mimic or scramble with SKA2 3′UTR-Wt or SKA2 3′UTR-Mut plasmid to U87 and U251 glioma cells, luciferase activity was analyzed. The results showed that the relative luciferase activity of the plasmid carrying SKA2 3′UTR-WT was significantly suppressed in the presence of miR-141 mimic. In contrast, this effect was not detected in the plasmid carrying SKA2 3′UTR -Mut (Figure 5B–5C). We further examined SKA2 mRNA and protein expression in U87 and U251 cells after transfection of miR-141 mimic. We found that miR-141 mimic reduced SKA2 mRNA and protein expression (Figure 5D–5F). To explore whether miR-141 exerts biological functions by SKA2, we performed a rescue experiment. Data from the MTT assay showed that miR-141 inhibitor promoted and si-SKA2 suppressed glioma cell proliferation. Co-transfection of miR-141 inhibitor and si-SKA2 showed that miR-141 inhibitor increased cell proliferation suppressed by si-SKA2 (Figure 6A–6B). Clone formation assay demonstrated that miR-141 knockdown promoted clone formation in glioma cells, while cotransfection with si-SKA2 moderated the effect of miR-141 knockdown (Figure 6C). In addition, the invasion was promoted in miR-141 inhibitor treated glioma cells, while SKA2 knockdown reversed the invasion mediated by miR-141 inhibitor (Figure 6D). Therefore, these results suggest that miR-141 acts its tumor suppressor roles by SKA2 in glioma cells.

### HOTAIR regulates the miR-141 target, SKA2

**Figure 7:**
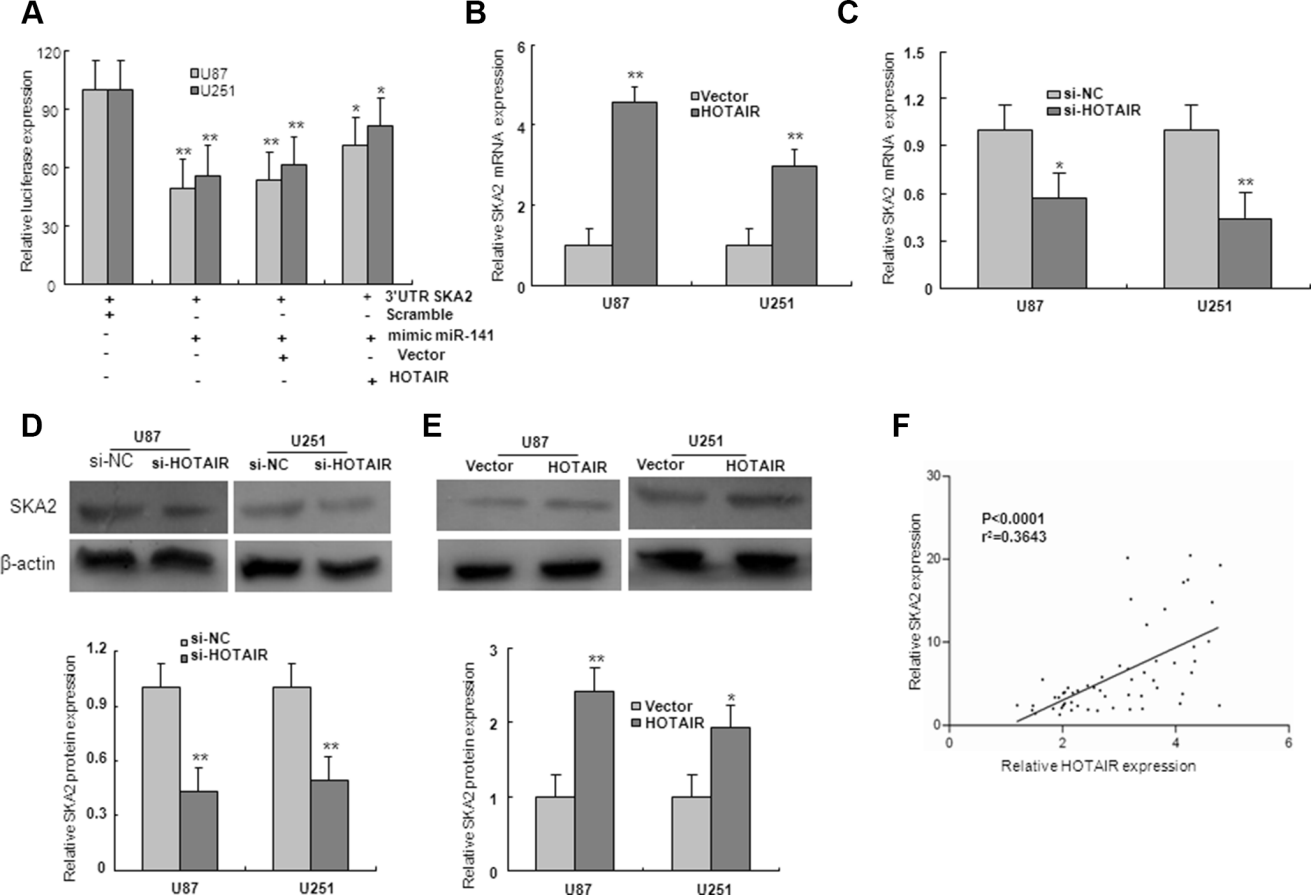
HOTAIR regulate miR-141 target, SKA2 (**A**) The 3′UTR of SKA2 was fused to the luciferase coding region (psiCHECK-SKA2 3′UTR) and transfected in U87 and U251 glioma cells with miR-141mimic to verify SKA2 is the target of miR-141. psiCHECK-SKA2 3′UTR and miR-141 mimic constructs were co-transfected into cells with plasmids expressing HOTAIR or with a control vector to confirm the ceRNA activity of HOTAIR. (**B**–**E**) The level of SKA2 mRNA and protein expression in U87 and U251 glioma cells upon HOTAIR, si-HOTAIR or control transfection. (**F**) The correlation between SKA2 and HOTAIR mRNA level was measured in 56 glioma tissues. The ΔCt values were subjected to Pearson correlation analysis. The data represent the mean ± SD of three different experiments. *P < 0.05, **P < 0.01 versus Normal or scramble control.

To examine if HOTAIR is involved in regulation of miR-141 target genes, the SKA2 3′UTR construct was subsequently transfected together with HOTAIR and miR-141 mimic in U87 and U251 glioma cells. Luciferase assays indicated that, in the presence of HOTAIR, miR-141 mediated-SKA2 3′UTR repression was restored compared with the control group (Figure 7A). To investigate whether HOTAIR regulated miR-141 target gene SKA2, U87 and U251 cells were transfected with HOTAIR or si-HOTAIR. The expression levels of SKA2 mRNA and protein were markedly downregulated by transfection of si-HOTAIR, and upregulated by overexpression of HOTAIR (Figure 7B–7E). Because HOTAIR could upregulate SKA2, we next examined whether HOTAIR was co-expressed with SKA2 in human glioma samples. We measured the expression levels of HOTAIR and SKA2 in 56 human glioma tissues. As shown in Figure 7F, HOTAIR transcript level was positively correlated with SKA2 mRNA level. Together, these results suggest that HOTAIR may act as a competing endogenous RNA (ceRNA) for the target SKA2 through binding miR-141, thereby regulating the derepression of SKA2.

### Effect of DNA methylation on miR-141 expression

**Figure 8:**
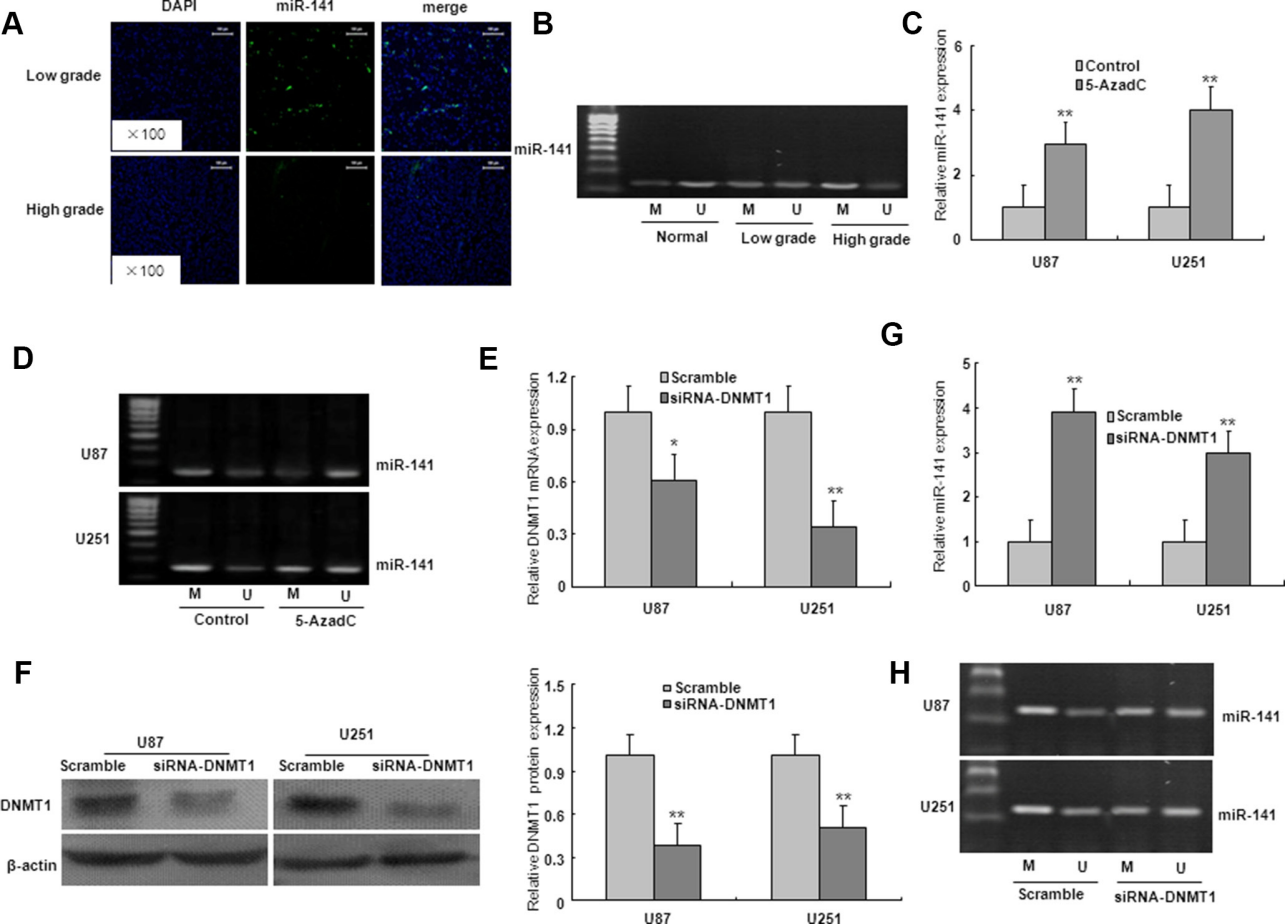
The down-regulation of miR-141 due to the hypermethylation of its promoter region in glioma (**A**). ISH with anti-miR-141 probe was performed to determine the localization of miR-141 in low grade glioma and high grade glioma. Representative views are presented (×100). (**B**) miR-141 methylation status in normal brain tissues, low grade glioma tissues, and high grade glioma tissues. (**C**) Effects of 5-azadC on miR-141 expression in U87 and U251 glioma cells. (**D**) Effects of 5-azadC on miR-141 promoter methylation in U87 and U251 glioma cells. (**E**–**F**) The mRNA and protein expression of DNMT1 was measured in DNMT1 siRNA-transfected U87 and U251 glioma cells. (**G**) The mRNA expression of miR-141 was analyzed in DNMT1 siRNA-transfected U87 and U251 glioma cells. (**H**) miR-141 promoter methylation was measured in DNMT1 siRNA-transfected U87 and U251 glioma cells. The data represent the mean ± SD of three different experiments. *P < 0.05, **P < 0.01 versus Control or scramble group.

We investigated whether the downregulation of miR-141 expression in glioma was caused by an epigenetic mechanism. As shown in Figure 8A, the miR-141 was localized in the cellular nucleus and cytoplasm in low grade glioma tissues, however, high grade glioma tissues lacked miR-141 expression. The MSP result showed that compared with normal brain tissues, the promoter CpG islands of miR-141 were hypermethylated in low grade and high glioma specimens which miR-141 expression was downregulated, strongly suggesting an essential role of promoter methylation in miR-141 down-regulation (Figure 8B).

To further validate this finding, we treated the cells with DNA methyltransferase inhibitor 5-AzadC for 48 h. Compared with the controls, the expression of miR-141 was significantly upregulated, and the demethylation of miR-141 in U251 and U87 glioma cells with 5-AzadC treatment (Figure 8C–8D). These findings suggest that the downregulation of miR-141 may be due to the hypermethylation of miR-141 promoter. To determine whether DNMT1 is involved in the methylation of miR-141 in glioma cells, the expression of DNMT1 gene was inhibited by DNMT1 RNAi (Figure 8E–8F). As illustrated in Figure 7G–7H, DNMT1 knockdown with RNAi in U251 and U87 glioma cells ameliorated miR-141 methylation, and restored miR-141 mRNA expression. These results suggest that the loss of miR-141 is dependent on DNMT1 in glioma cells.

### Both overexpression of miR-141 and knockdown of HOTAIR inhibit xenograft tumor growth *in vivo*

**Figure 9:**
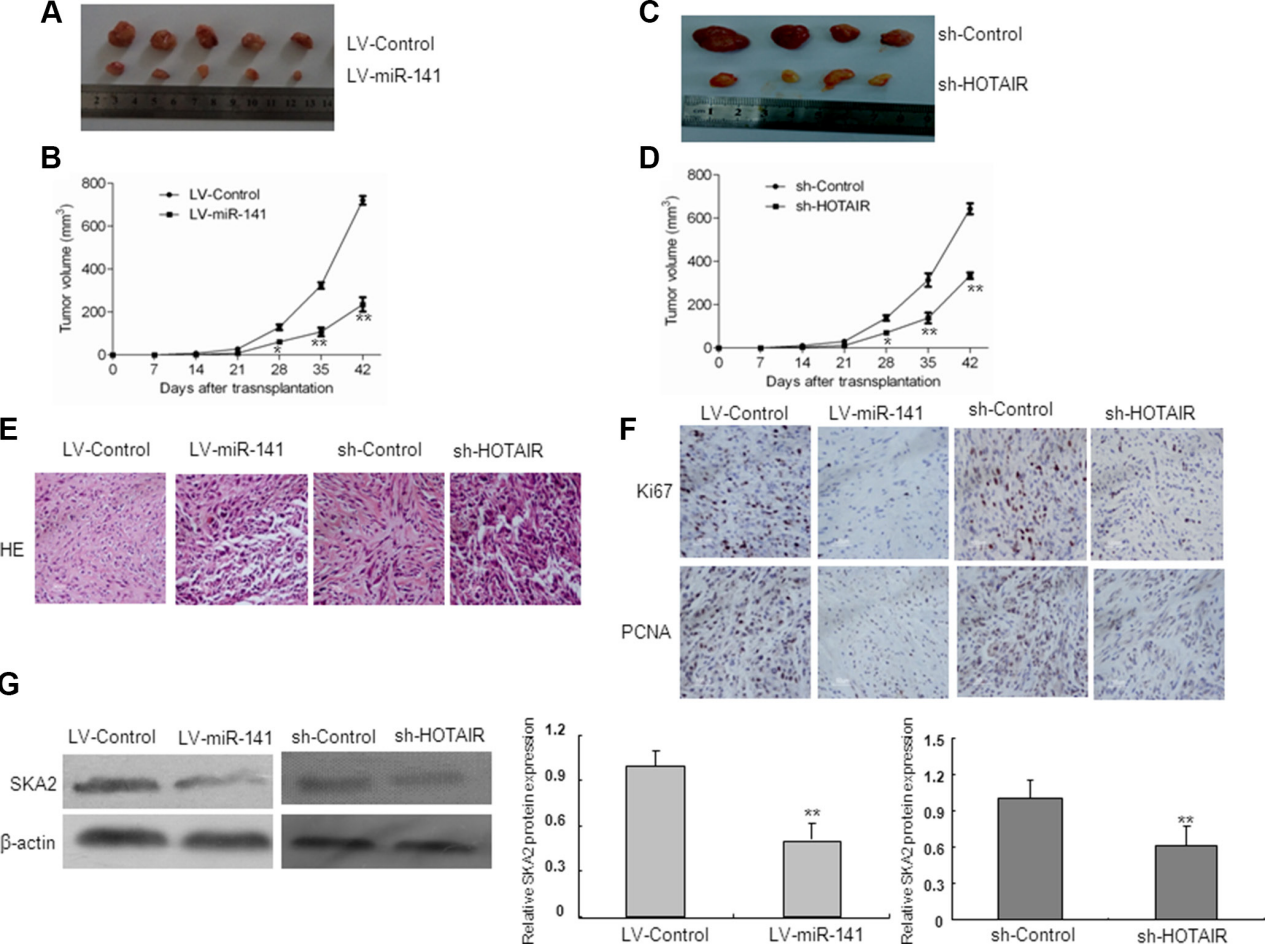
In *vivo* tumor xenografts study Nude mice were subcutaneously injected with LV-miR-141 or sh-HOTAIR transfected U87 cells. (**A**) Sample tumors from LV-miR-141 and sh-HOTAIR were shown. (**B**) Tumor volume was calculated every 7 days after injection. (**C**) Hematoxylin and eosin staining of xenograft tumor tissues. Magnification, 40×. (**D**) Ki67 and PCNA expression was detected by immunohistochemistry assay in xenograft tumor sections, Magnification, 40×. (E) SKA2 protein expression in LV-miR-141 tumors and sh-HOTAIR tumors. The data represent the mean ± SD of three different experiments. **P < 0.01 versus LV-control.

To test the function of miR-141 *in vivo*, we established a xenograft tumor model in nude mice with LV-miR-141 and sh-HOTAIR infected U87 glioma cells. As shown in Figure 9A–9D, both LV-miR-141 and sh-HOTAIR significantly reduced xenograft tumor growth. H&E staining showed decreased cell density in LV-miR-141 and sh-HOTAIR xenografts (Figure 9E). Next, immunostaining analysis of the proliferation marker PCNA and Ki67 was performed in resected tumor tissues. The number of PCNA and Ki67 positive cells significantly reduced in the LV-miR-141 and sh-HOTAIR xenograft tumor tissues (Figure 9F). Western blot analyses showed that SKA2 expression was decreased in the LV-miR-141 and sh-HOTAIR xenograft tumor (Figure 9E). These results suggest that miR-141 reduces the *in vivo* proliferation capacity of glioma cells, which is associated with HOTAIR.

## DISCUSSION

Aberrant expression of miRNAs is closely related to tumorigenesis. Recent data have shown that miR-141 plays important roles in tumorigenesis. For example, miR-141 has been found to be up-regulated in ovarian cancer and colon cancer, and acts as an oncogene [24, 25]. While the other studies show that miR-141 is downregulated in pancreatic cancer, renal cell carcinoma and gastric cancer [26–28]. However, the function of miR-141 in gliomas has not yet been elucidated. In the present study, we demonstrated that miR-141 expression was significantly downregulated in gliomas tissues and correlated with pathological grade of gliomas. Recently, Liu et al. reported that miR-141 inhibited malignant biological phenotype of hepatocellular carcinoma cells, but in contrast to other studies that suggested miR-141 acted as an oncogene in nasopharyngeal carcinoma [29, 30]. Here, we demonstrated that miR-141 significantly suppressed glioma cell proliferation, migration and invasion, whereas inhibition of miR-141 exerted the opposite effect. These observations imply that miR-141 may be a tumor suppressor miRNA and therapeutic target in gliomas.

Recently, increasing studies have showed that miRNAs have an effect on the regulation of lncRNAs. MiR-34a binds directly to HOTAIR and represses expression levels of HOTAIR in prostate cancer cells [31]. In addition, HOTAIR promotes cell cycle progression in glioma as a result of the binding of its 5′ domain to the PRC2 complex [32]. Chiyomaru et al reported that HOTAIR is one of the downstream targets of miR-141 in renal carcinoma cells [23]. Here, we presented strong evidence that miR-141 could inhibit the expression of HOTAIR by combining directly to the 3′UTR of HOTAIR in glioma cells, and an inverse correlation between miR-141 and HOTAIR expression in glioma tissues. Furthermore, our analyses demonstrated that miR-141 inhibited the proliferation and invasiveness of glioma cells, whereas HOTAIR reversed the effects that miRNA-141 exerted. In accordance with the fact that HOTAIR promotes malignancy, whereas miR-141 suppresses malignancy in a variety of tumors [33, 34]. These results suggest that HOTAIR is involved in miR-141-mediated proliferation and invasion potential in glioma.

Spindle and kinetochore associated complex subunit 2(SKA2) located on human chromosome 17q 23.2 is involved in the maintenance of the metaphase plate and/or spindle checkpoint silencing [35]. Recent evidences showed that overexpression of SKA2 promoted proliferation and human breast cancer progression, whereas SKA2 knockdown in human lung epithelial cells reduced transactivation and suppressed dexamethasone inhibition of proliferation [36, 37]. In an approach using a combination of in silico miRNA target prediction and target confirmation by 3′UTR luciferase assays, qRT-PCR and western blotting, we identified SKA2 as a novel target of miR-141 in human glioma cells. We further demonstrated that miR-141 inhibited the proliferation, migration and invasion by targeting the 3′-UTR of SKA2 in human glioma cells. *In vivo* experiments also confirmed that miR-141 inhibited xenograft tumor growth by targeting SKA2.

Recently, increasing studies have showed that lncRNAs may act as endogenous sponge RNAs to interact with miRNAs by competing with miRNAs for binding to target mRNAs, and then to silence target genes. The miRNA associated with lncRNA was firstly put forward in 2007, a study from Arabidopsis thaliana that found an lncRNA IPS1 to bind to the miR-399, and then repressed its target mRNA [38]. A recent report showed that highly up-regulated liver cancer (HULC) might act as an endogenous ‘sponge’ of miR-372, which results in reducing translational repression of its target gene [39]. To investigate the miRNA-related functions of HOTAIR in glioma, we chose miR-141 as a model miRNA for further studies, with a particular focus on the target gene SKA2. In this study, luciferase assays confirmed the existence of specific crosstalk between HOTAIR and SKA2 through competition for miR-141 binding. Consistent with HOTAIR sequestration of miR-141, showed that HOTAIR actually regulated SKA2 protein expression both *in vitro* and *in vivo*. These results are similar with previous report that a competing endogenous RNA HOTAIR is transmodulators of gene expression through competing miR-331-3p binding [40]. The positive correlation between SKA2 and HOTAIR expression and the relevance to miR-141 expression levels supports our hypothesis that ceRNA can sequester miRNAs, thereby protecting their target mRNAs from repression.

CpG islands hypermethylation in the promoter region of specific genes was firmly established as an important regulatory mechanism for the inactivation of those genes [41]. MiRNA deregulation can occur at both the transcriptional and processing level, and aberrant DNA methylation patterns have been implicated in altering miRNA expression in the cancer development process [42–44]. DNA methylation is associated with repression of miRNAs possessing promoter-associated CpG islands. Recently, increasing studies have reported that DNA methylation of tumor-suppressor miRNAs, including miR-211, miR-23b and miR-145, is observed in glioma [45–47]. We showed that miR-141 hypermethylation was found in high grade glioma tissues. The expressions and functions of these tumor-suppressor miRNAs can be reversed and restored by the demethylating agent 5-AzadC [48]. In the present study, we demonstrated that 5-AzadC treatment induced the demethylation of miR-141, and restored miR-141 expression in glioma cells. As we know, DNA methylation is established and maintained by DNMTs and is frequently disrupted in cancers, thus contributes directly and indirectly to carcinogenesis [49]. Here, we showed that DNMT1 was involved in epigenetic repression of miR-141 in glioma cells. These findings suggest that the loss of miR-141 expression probably attributed, at least in part, to epigenetic modification by the hypermethylation of miR-141 promoter in gliomas.

**Figure 10:**
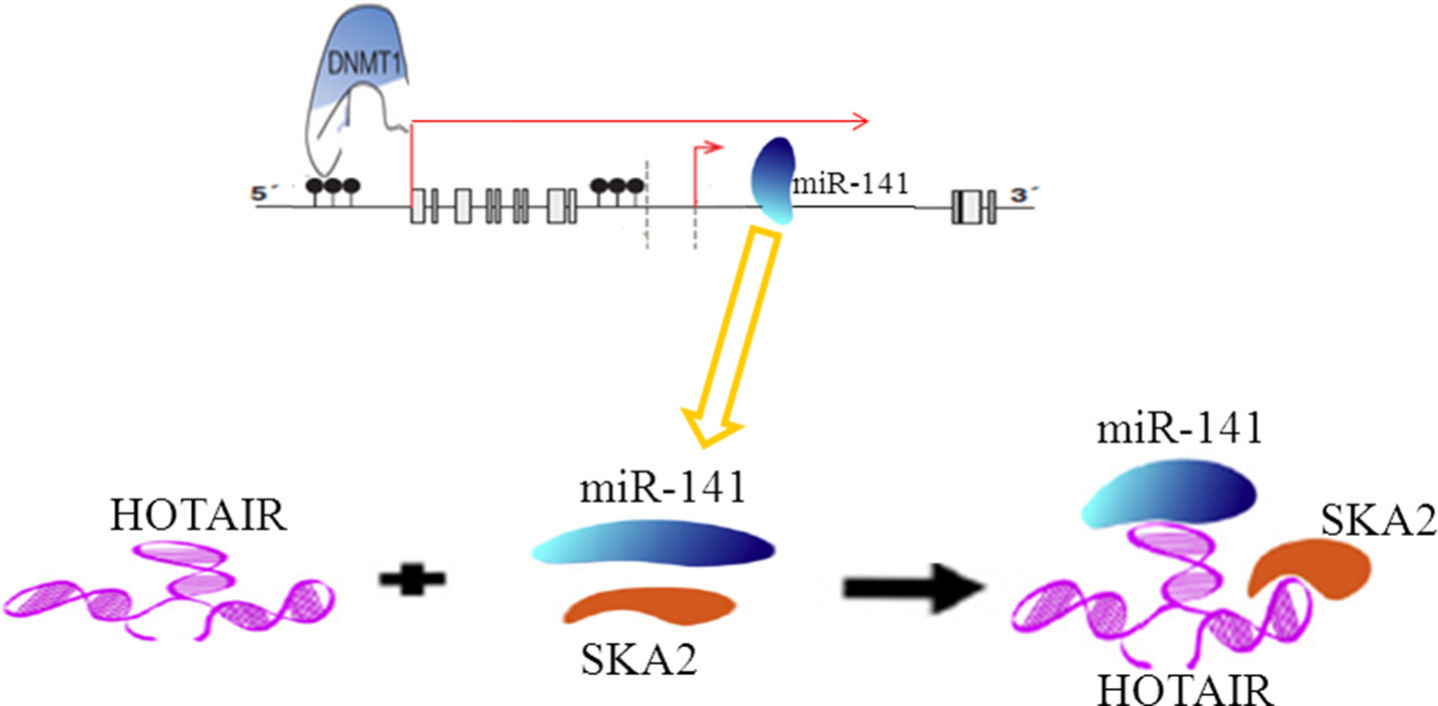
A schematic model of methylation of miR-141 mediated by DNMT1 regulates SKA2 by an endogenous ‘sponge’ HOTAIR in glioma

In conclusion, our findings from the present study strongly suggest the involvement of HOTAIR in abnormal expression of miR-141 mediated by epigenetic modification targeted SKA2 in glioma (Figure 10). MiR-141 as a candidate tumor suppressor miRNA related to the pathogenesis and progression of human gliomas. HOTAIR may act as an endogenous ‘sponge’ by binding miR-141, thereby abolishing the miRNA-induced repression of SKA2. Moreover, Epigenetic inactivation of miR-141 is a common event in glioma. It is worth mentioning that HOTAIR may act as endogenous sponge RNA and sequester a handful of miRNAs, while miR-141 is also able to regulate multiple targets. Thus, the multiple properties of HOTAIR are possibly due to simultaneous targeting of multiple targets in glioma. Further investigation of non-coding RNA could therefore provide more information related to the pathogenesis of human gliomas; reveal novel mechanisms to broaden our knowledge of the involvement of epigenetic modification in human gliomas; and eventually provide new therapeutic strategies with gliomas.

## MATERIALS AND METHODS

### Cell lines and patient tissue samples

Human glioma cell lines U87, U251 and A172 were purchased from Cell Bank of Type Culture Collection of Chinese Academy of Sciences (Shanghai, China). Dulbecco's modified Eagle's medium (DMEM) and fetal bovine serum (FBS) were obtained from Hyclone (Logan, Utah, USA). Tissue samples were collected from the neurosurgery department of The Second Affiliated Hospital of Anhui Medical University (HeFei, China). Samples were collected and snap-frozen in liquid nitrogen immediately and preserved in −80°C. A total of 67 samples were used for this study including (WHO I/II, *n* = 31), (WHO III/IV, *n* = 25) and normal brain tissues derived from the temporal lobes and saddle area of the patients with arachnoid cyst after surgery (*n* = 11), both glioma patients and controls had the similar percentages with respect to sex and age. This study was approved by the Research Ethnics Committee of The Second Affiliated Hospital of Anhui Medical University. Informed consent was obtained from all patients.

### 5-aza-2′-deoxycytidine treatment

U87 and U251 cells were cultured overnight, 5-aza-2′-deoxycytidine (5-AzadC) was added and was refreshed every 24 h until 48 h treatment finished. The medium containing PBS only was regarded as a control.

### Methylation-specific polymerase chain reaction

The methylation status of the miR-141 promoter region was determined by methylation-specific PCR (MSP) using bisulfite-modified DNA. Two primer sets were used to amplify the promoter region of the miR-141 that incorporated a number of CpG sites, one specific for the methylated sequence (miR-141-M, forward:5′-TTCGGGAGTAGTTCGGTTC-3′;reverse:5′-AATTAAACTATACCGCCCCG -3′) and the other for the unmethylated sequence (miR-141-U, forward: 5′-GGTTTGGGAGTAGTTTGGTTT-3′;reverse:5′-AAAT TAAACTATACCACCCCAC -3′). MSP experiments were performed at least in duplicate.

### RNA interference (RNAi) analysis and plasmid construction

Small interfering RNA (siRNA) and nonspecific control siRNA was synthesized (Genepharma, Shanghai, China) and transfected using Lipofectamine 2000 (Invitrogen, Carlsbad, CA). The HOTAIR sequences were synthesized and subcloned into the pCDNA3.1 vector. The pCDNA constructs or the empty vector were transfected into glioma cells cultured on six-well plates according to the manufacturer's instructions. The sequences of the siRNAs are described in Supplementary File.

### Real-time PCR analysis

Total RNA was extracted from glioma specimens or glioma cells using TRIzol reagent (Invitrogen). Real-time quantitative PCR analysis was performed using SYBR Green Master Mix Kit on Thermo Fisher connect Real-Time PCR platform. For relative quantification, 2^−ΔΔCT^ was calculated and used as an indication of the relative expression levels, which was calculated by subtracting CT values of the control gene from the CT values of miR-141, HOTAIR, SKA2 and DNMT1. Real time PCR was carried out under a standard protocol using the following primers(Supplementary File).

### Cell proliferation

Cells were seeded into 96-well plate with a concentration of 3000 cells per well, and incubated at 37°C 4 days post infection. The number of viable cells was measured at daily intervals (hour 24, 48, 72 and 96). At each time point, 10 μl of 5 mg/ml MTT (Dingguo Biotechnology) was added, and incubation was continued for 4 h. Then the medium was removed carefully and 150 μl of DMSO was added at the end of incubation. The absorbance was measured at 592 nm on the spectrophotometer. For the colony formation assay, a total of 200 cells were seeded in 6-well plates in triplicate and maintained in the complete medium for 10 days. The natural colonies were washed with PBS and fixed with 4% paraformaldehyde for 30 min at room temperature. The colonies were then stained with Giemsa for 10 min, washed with water and air-dried. The total number of colonies with more than 50 cells was counted under fluorescence microscopy.

### Scratch wound assay

Glioma cells were transfected with miR-141 mimic, miR-141 inhibitor or si-NC. Wounds were created in adherent cells using a 20 μL pipette tip, 24 h after transfection. The cells were then washed three times with PBS to remove any free-floating cells and debris. Medium without serum was added, and the cells were incubated under normal conditions. Wound healing was observed after 24 h under light microscopy. Representative scrape lines were photographed using digital microscopy after culture inserts were removed. Each experiment was repeated in triplicate.

### *In vitro* migration and invasion assays

Glioma cells (1 × 10^5^) were plated on the top side of polycarbonate Transwell filters (without Matrigel for Transwell assay) or plated on the top side of polycarbonate Transwell filter coated with Matrigel (for Transwell matrix penetration assay) in the upper chamber of the QCM^™^ 24-Well Cell Invasion Assay (Cell Biolabs, INC, USA). For migration assays, cells were suspended in medium without serum, and medium without serum was used in the lower chamber. For the invasion assay, cells were suspended in medium without serum, and medium supplemented with serum was used as a chemoattractant in the lower chamber. The cells were incubated at 37°C for 24 hours (migration assay) or (invasion assay). The non-migratory or non-invasive cells in the top chambers were removed with cotton swabs. The migrated and invaded cells on the lower membrane surface were fixed with Methanol and stained with crystal violet. Cells were counted visually in 5 random fields under light microscope (10 × objective lens). In addition, migrated and invaded cells were dissociated, lysed and quantified at 570 nm using spectrophotometer.

### Western blotting

Cells were lysed with RIPA lysis buffer (Beyotime, China). Whole-cell extracts (20 or 40 μg) were then fractionated by electrophoresis through an 8% or 12% sodium dodecyl sulfate-polyacrylamide gel electrophoresis (SDS-PAGE). Gels were run at a 120 V for 2 h before transfer onto a PVDF membrane (MilliporeCorp, Billerica, MA, U.S.A.). Anti-β-actin, Anti-DNMT1, anti-SKA2 were diluted 1:1000. Following incubation with the primary antibody, blots were washed three times in TBS/Tween-20 before incubation for 1 h with goat anti-mouse or mouse anti-rabbit horseradish peroxidase conjugated antibody at a 1:10000 dilution in TBS/Tween-20 containing 5% milk. Proteins were visualized with ECL-chemiluminescent kit (ECL-plus, Thermo Scientific).

### Luciferase assay

Human glioma cells (1 × 10^4^) grown in a 96-well plate were co-transfected with 150 ng of the corresponding psiCHECK2 plasmid, 50 nM miR-141 mimic or mimic NC, and comprising 3′UTR of SKA2, wild type or mutant HOTAIR or SKA2 fragment using Lipofectamine 2000 (Invitrogen, USA) as the transfection reagent. Cells were harvested 48 h after transfection for luciferase assay using a luciferase assay kit (Promega) according to the manufacturer's protocol. The values were normalized to those obtained for miRNA negative control transfection. All transfection experiments were performed in triplicate.

### Fluorescence *in situ* hybridization (FISH) detection

*In situ* hybridisation (ISH) was performed with an *in situ* hybridization kit (Boster Biological Technology, Ltd., Wuhan, China). The glioma tissue microarrays were deproteinated, and then prehybridized for 2 h in hybridization liquid in a humidified chamber (50% formamide, 5 × SSC). MiR-141 probes were added to the sections on the microarray and incubated overnight at 40°C in a water bath. The anti-digoxigenin-rhodamine and streptavidin-FITC solution was added and incubated for 2 h at room temperature in the dark. Nuclei were counterstained with a DAPI karyotyping kit (Genmed, USA). Sections were sealed and detected under a fluorescence microscope with an OptiGrid system and analyzed by IPP6.1 (Olympus, Tokyo, Japan).

### Tumor formation study *in vivo*

Tumor formation was studied by establishing a xenograft model. Lentiviral vectors(GeneChemCo. Ltd, Shanghai, China) containing miR-141 (LV-miR-141) and sh-HOTAIR were used to infect U87 glioma cells according to the manufacturer's instructions. BALB/c female nude mice (4 weeks old) were purchased from Beijing HFK Bio-Technology Co., Ltd (China). The animal experiments in this study were approved and reviewed by the Animal Research Committee of Anhui Medical University. Care and handling of the animals were in accordance with the guidelines for Institutional and Animal Care and Use Committees. Mice were randomly divided into 2 groups with 6 mice in each group. Infected U87 glioma (5 × 10^6^ cells/mouse) suspended in PBS solution were injected subcutaneously into the mice. Tumor volumes were measured every 7 days using calipers along two major axes and calculated according to the formula V = 0.5 × L (length) × W_2_ (width). At 42 days after cell inoculation, mice were sacrificed. Excised tumors were evaluated for volume and weight.

### Statistical analysis

All data were expressed as mean ± SD of three independent experiments, in which each assay was performed in triplicate. Statistical analysis was performed using ANOVA followed by Student's *t*-test. The relationship between the expression of HOTAIR, SKA2 and miR-141 in tissues was analyzed with Pearson's correlation. Significance was defined as *P* < 0.05.

## SUPPLEMENTARY MATERIALS



## Abbreviations

GBM: Glioblastoma multiforme
microRNA: miRNA
3′-UTR: 3′-untranslated regions
LncRNAs: Long non-coding RNAs
HOTAIR: Hox transcript antisense intergenic RNA
SKA2: spindle and kinetochore associated complex subunit 2
HULC: up-regulated liver cancer
5-AzadC: 5-aza-2′-deoxycytidine
ceRNA: competing endogenous RNA

## References

[1] Wang Y, Jiang T (2013). Understanding high grade glioma: molecular mechanism, therapy and comprehensive management. Cancer Lett.

[2] Adamson C, Kanu OO, Mehta AI, Di C, Lin N, Mattox AK, Bigner DD (2009). Glioblastoma multiforme: a review of where we have been and where we are going. Expert Opin Investig Drugs.

[3] Drappatz J, Norden AD, Wen PY (2009). Therapeutic strategies for inhibiting invasion in glioblastoma. Expert Rev Neurother.

[4] Bartel DP (2004). MicroRNAs: genomics, biogenesis, mechanism, and function. Cell.

[5] Winter J, Jung S, Keller S, Gregory RI, Diederichs S (2009). Many roads to maturity: microRNA biogenesis pathways and their regulation. Nat Cell Biol.

[6] Bracken CP, Gregory PA, Khew-Goodall Y, Goodall GJ (2009). The role of microRNAs in metastasis and epithelial-mesenchymal transition. Cell Mol Life Sci.

[7] Negrini M, Nicoloso MS, Calin GA (2009). MicroRNAs and cancer--new paradigms in molecular oncology. Curr Opin Cell Biol.

[8] Ma J, Yao Y, Wang P, Liu Y, Zhao L, Li Z, Xue Y (2014). MiR-152 functions as a tumor suppressor in glioblastoma stem cells by targeting Kruppel-like factor 4. Cancer Lett.

[9] Shi Y, Luo X, Li P, Tan J, Wang X, Xiang T, Ren G (2015). miR-7-5p suppresses cell proliferation and induces apoptosis of breast cancer cells mainly by targeting REGgamma. Cancer Lett.

[10] Menigatti M, Staiano T, Manser CN, Bauerfeind P, Komljenovic A, Robinson M, Jiricny J, Buffoli F, Marra G (2013). Epigenetic silencing of monoallelically methylated miRNA loci in precancerous colorectal lesions. Oncogenesis.

[11] Seol HS, Akiyama Y, Shimada S, Lee HJ, Kim TI, Chun SM, Singh SR, Jang SJ (2014). Epigenetic silencing of microRNA-373 to epithelial-mesenchymal transition in non-small cell lung cancer through IRAK2 and LAMP1 axes. Cancer Lett.

[12] Li X, Roslan S, Johnstone CN, Wright JA, Bracken CP, Anderson M, Bert AG, Selth LA, Anderson RL, Goodall GJ, Gregory PA, Khew-Goodall Y (2014). MiR-200 can repress breast cancer metastasis through ZEB1-independent but moesin-dependent pathways. Oncogene.

[13] Roy SS, Gonugunta VK, Bandyopadhyay A, Rao MK, Goodall GJ, Sun LZ, Tekmal RR, Vadlamudi RK (2014). Significance of PELP1/HDAC2/miR-200 regulatory network in EMT and metastasis of breast cancer. Oncogene.

[14] Flintoft L (2013). Non-coding RNA:Structure and function for lncRNAs. Nat Rev Genet.

[15] Mercer TR, Dinger ME, Mattick JS (2009). Long non-coding RNAs: insights into functions. Nat Rev Genet.

[16] Guttman M, Donaghey J, Carey BW, Garber M, Grenier JK, Munson G, Young G, Lucas AB, Ach R, Bruhn L, Yang X, Amit I, Meissner A (2011). lincRNAs act in the circuitry controlling pluripotency and differentiation. Nature.

[17] Tan JY, Sirey T, Honti F, Graham B, Piovesan A, Merkenschlager M, Webber C, Ponting CP, Marques AC (2015). Extensive microRNA-mediated crosstalk between lncRNAs and mRNAs in mouse embryonic stem cells. Genome Res.

[18] Kogo R, Shimamura T, Mimori K, Kawahara K, Imoto S, Sudo T, Tanaka F, Shibata K, Suzuki A, Komune S, Miyano S, Mori M (2011). Long noncoding RNA HOTAIR regulates polycomb-dependent chromatin modification and is associated with poor prognosis in colorectal cancers. Cancer Res.

[19] Geng YJ, Xie SL, Li Q, Ma J, Wang GY (2011). Large intervening non-coding RNA HOTAIR is associated with hepatocellular carcinoma progression. J Int Med Res.

[20] Sorensen KP, Thomassen M, Tan Q, Bak M, Cold S, Burton M, Larsen MJ, Kruse TA (2013). Long non-coding RNA HOTAIR is an independent prognostic marker of metastasis in estrogen receptor-positive primary breast cancer. Breast Cancer Res Treat.

[21] Svoboda M, Slyskova J, Schneiderova M, Makovicky P, Bielik L, Levy M, Lipska L, Hemmelova B, Kala Z, Protivankova M, Vycital O, Liska V, Schwarzova L (2014). HOTAIR long non-coding RNA is a negative prognostic factor not only in primary tumors, but also in the blood of colorectal cancer patients. Carcinogenesis.

[22] Zhang JX, Han L, Bao ZS, Wang YY, Chen LY, Yan W, Yu SZ, Pu PY, Liu N, You YP, Jiang T, Kang CS (2013). HOTAIR, a cell cycle-associated long noncoding RNA and a strong predictor of survival, is preferentially expressed in classical and mesenchymal glioma. Neuro Oncol.

[23] Chiyomaru T, Fukuhara S, Saini S, Majid S, Deng G, Shahryari V, Chang I, Tanaka Y, Enokida H, Nakagawa M, Dahiya R, Yamamura S (2014). Long non-coding RNA HOTAIR is targeted and regulated by miR-141 in human cancer cells. J Biol Chem.

[24] Iorio MV, Visone R, Di Leva G, Donati V, Petrocca F, Casalini P, Taccioli C, Volinia S, Liu CG, Alder H, Calin GA, Menard S, Croce CM (2007). MicroRNA signatures in human ovarian cancer. Cancer Res.

[25] Schetter AJ, Leung SY, Sohn JJ, Zanetti KA, Bowman ED, Yanaihara N, Yuen ST, Chan TL, Kwong DL, Au GK, Liu CG, Calin GA, Croce CM (2008). MicroRNA expression profiles associated with prognosis and therapeutic outcome in colon adenocarcinoma. JAMA.

[26] Du Y, Xu Y, Ding L, Yao H, Yu H, Zhou T, Si J (2009). Down-regulation of miR-141 in gastric cancer and its involvement in cell growth. J Gastroenterol.

[27] Gregory PA, Bert AG, Paterson EL, Barry SC, Tsykin A, Farshid G, Vadas MA, Khew-Goodall Y, Goodall GJ (2008). The miR-200 family and miR-205 regulate epithelial to mesenchymal transition by targeting ZEB1 and SIP1. Nat Cell Biol.

[28] Nakada C, Matsuura K, Tsukamoto Y, Tanigawa M, Yoshimoto T, Narimatsu T, Nguyen LT, Hijiya N, Uchida T, Sato F, Mimata H, Seto M, Moriyama M (2008). Genome-wide microRNA expression profiling in renal cell carcinoma: significant down-regulation of miR-141 and miR-200c. J Pathol.

[29] Liu Y, Ding Y, Huang J, Wang S, Ni W, Guan J, Li Q, Zhang Y, Chen B, Chen L (2014). MiR-141 suppresses the migration and invasion of HCC cells by targeting Tiam1. PLoS One.

[30] Zhang L, Deng T, Li X, Liu H, Zhou H, Ma J, Wu M, Zhou M, Shen S, Niu Z, Zhang W, Shi L, Xiang B (2010). microRNA-141 is involved in a nasopharyngeal carcinoma-related genes network. Carcinogenesis.

[31] Chiyomaru T, Yamamura S, Fukuhara S, Yoshino H, Kinoshita T, Majid S, Saini S, Chang I, Tanaka Y, Enokida H, Seki N, Nakagawa M, Dahiya R (2013). Genistein inhibits prostate cancer cell growth by targeting miR-34a and oncogenic HOTAIR. PLoS One.

[32] Zhang K, Sun X, Zhou X, Han L, Chen L, Shi Z, Zhang A, Ye M, Wang Q, Liu C, Seki N, Nakagawa M, Dahiya R (2015). Long non-coding RNA HOTAIR promotes glioblastoma cell cycle progression in an EZH2 dependent manner. Oncotarget.

[33] Chen FJ, Sun M, Li SQ, Wu QQ, Ji L, Liu ZL, Zhou GZ, Cao G, Jin L, Xie HW, Wang CM, Lv J, De W (2013). Upregulation of the long non-coding RNA HOTAIR promotes esophageal squamous cell carcinoma metastasis and poor prognosis. Mol Carcinog.

[34] Liu XH, Liu ZL, Sun M, Liu J, Wang ZX, De W (2013). The long non-coding RNA HOTAIR indicates a poor prognosis and promotes metastasis in non-small cell lung cancer. BMC Cancer.

[35] Hanisch A, Sillje HH, Nigg EA (2006). Timely anaphase onset requires a novel spindle and kinetochore complex comprising Ska1 and Ska2. EMBO J.

[36] Rice L, Waters CE, Eccles J, Garside H, Sommer P, Kay P, Blackhall FH, Zeef L, Telfer B, Stratford I, Clarke R, Singh D, Stevens A (2008). Identification and functional analysis of SKA2 interaction with the glucocorticoid receptor. J Endocrinol.

[37] Shi W, Gerster K, Alajez NM, Tsang J, Waldron L, Pintilie M, Hui AB, Sykes J, P'ng C, Miller N, McCready D, Fyles A, Liu FF (2011). MicroRNA-301 mediates proliferation and invasion in human breast cancer. Cancer Res.

[38] Franco-Zorrilla JM, Valli A, Todesco M, Mateos I, Puga MI, Rubio-Somoza I, Leyva A, Weigel D, Garcia JA, Paz-Ares J (2007). Target mimicry provides a new mechanism for regulation of microRNA activity. Nat Genet.

[39] Wang J, Liu X, Wu H, Ni P, Gu Z, Qiao Y, Chen N, Sun F, Fan Q (2010). CREB up-regulates long non-coding RNA, HULC expression through interaction with microRNA-372 in liver cancer. Nucleic Acids Res.

[40] Liu XH, Sun M, Nie FQ, Ge YB, Zhang EB, Yin DD, Kong R, Xia R, Lu KH, Li JH, De W, Wang KM, Wang ZX (2014). Lnc RNA HOTAIR functions as a competing endogenous RNA to regulate HER2 expression by sponging miR-331-3p in gastric cancer. Mol Cancer.

[41] Gao W, Gu Y, Li Z, Cai H, Peng Q, Tu M, Kondo Y, Shinjo K, Zhu Y, Zhang J, Sekido Y, Han B, Qian Z (2015). miR-615-5p is epigenetically inactivated and functions as a tumor suppressor in pancreatic ductal adenocarcinoma. Oncogene.

[42] Han L, Witmer PD, Casey E, Valle D, Sukumar S (2007). DNA methylation regulates MicroRNA expression. Cancer Biol Ther.

[43] Lujambio A, Ropero S, Ballestar E, Fraga MF, Cerrato C, Setien F, Casado S, Suarez-Gauthier A, Sanchez-Cespedes M, Git A, Spiteri I, Das PP, Caldas C (2007). Genetic unmasking of an epigenetically silenced microRNA in human cancer cells. Cancer Res.

[44] Weber B, Stresemann C, Brueckner B, Lyko F (2007). Methylation of human microRNA genes in normal and neoplastic cells. Cell Cycle.

[45] Asuthkar S, Velpula KK, Chetty C, Gorantla B, Rao JS (2012). Epigenetic regulation of miRNA-211 by MMP-9 governs glioma cell apoptosis, chemosensitivity and radiosensitivity. Oncotarget.

[46] Geng J, Luo H, Pu Y, Zhou Z, Wu X, Xu W, Yang Z (2012). Methylation mediated silencing of miR-23b expression and its role in glioma stem cells. Neurosci Lett.

[47] Speranza MC, Frattini V, Pisati F, Kapetis D, Porrati P, Eoli M, Pellegatta S, Finocchiaro G (2012). NEDD9, a novel target of miR-145, increases the invasiveness of glioblastoma. Oncotarget.

[48] Momparler RL (2013). Epigenetic therapy of non-small cell lung cancer using decitabine (5-aza-2′-deoxycytidine). Front Oncol.

[49] Lopez-Bertoni H, Lal B, Li A, Caplan M, Guerrero-Cazares H, Eberhart CG, Quinones-Hinojosa A, Glas M, Scheffler B, Laterra J, Li Y (2014). DNMT-dependent suppression of microRNA regulates the induction of GBM tumor-propagating phenotype by Oct4 and Sox2. Oncogene.

