# Supplementary material for: Epigenetic modification of miR-141 regulates SKA2 by an endogenous ‘sponge’ HOTAIR in glioma
Source: Oncotarget. 2016 Apr 21;7(21):30610–25. doi: 10.18632/oncotarget.8895 (PMC5058705; doi:10.18632/oncotarget.8895)
Supplement: Supplementary file 1 [file oncotarget-07-30610-s001.pdf]

Epigenetic modification of miR-141 regulates SKA2 by an endogenous 'sponge' HOTAIR in glioma

Supplementary Materials

qRT-PCR primers

DNMT1 forward: 5′-CGGCTTCAGCACCTCATTTG-3′
reverse: 5′-AGGTCGAGTCGGAATTGCTC-3′
HOTAIR Forward: 5′-ACAGTGGCACCGCTTTTCTA-3
Reverse: 5′-GCAGGGTCCCACTGCATAAT-3
SKA2: Forward: 5′-CCGCTTTAAACCAGTTGCTG-3′,
Reverse: 5′-CTCTGCCGCAGTTTTCTCTT-3′.
GAPDH Forward: 5′-AGCAAGAGCACAAGAGGAAG-3′
Reverse: 5′-GGTTGAGCACAGGGTACTTT-3′.
miR-141 forward, 5′-CAUCUUCCAGUACAGUGUUGGA-3′
U6 forward, 5′-CTCGCTTCGGCAGCACA-3′

siRNA for HOTAIR, DNMT1 or SKA2

si-HOTAIR, 5′-AAUU-CUUAAAUUGGGCUGG-3′
si-DNMT1, 5′-GGGACUGUGUCUCUGUUAUTT-3′
5′-AUAACAGAGACACAGUCCCTT-3′
si-SKA2: 5′- AAGAAATCAAGACTAATCATCTT-3′.

To clone HOTAIR

sense: 5′-CATGGATCCACATTCTGCCCTGATTTCCGGAACC-3′
antisense: 5′-ACTCTCGAGCCACCACACACACACAACCTACAC-3′
